# Dynamic relationships among anxiety, psychological resilience, and academic engagement in junior secondary school students: a three-wave longitudinal study

**DOI:** 10.3389/fpsyg.2026.1885038

**Published:** 2026-07-28

**Authors:** Guoxing Bu, Yixuan Pang, Ye Li

**Affiliations:** Department of Education, Dongshin University, Naju-si, Republic of Korea

**Keywords:** academic engagement, anxiety, cross-lagged analysis, junior high school students, longitudinal mediation, psychological resilience

## Abstract

This study investigated the dynamic temporal relationships among anxiety, psychological resilience, and academic engagement among junior secondary school students, with particular attention to the longitudinal mediating role of psychological resilience in the relationship between anxiety and academic engagement. Using a three-wave longitudinal design, this study conducted a 6-month questionnaire survey at a general junior secondary school in eastern China. A total of 610 students completed the Screening for Childhood Anxiety and Depressive Disorders (SCARED), the Connor-Davidson Resilience Scale (CD-RISC), and the University of Washington Engagement Scale (UWES-S) at three time points. Cross-lagged panel models and longitudinal mediation models were used for statistical analysis. The results showed that: (1) anxiety and psychological resilience exhibited a bidirectional negative predictive relationship in the early phase, whereas in the later phase, only the protective effect of psychological resilience on anxiety remained significant; (2) anxiety consistently and unidirectionally negatively predicted subsequent academic engagement; (3) psychological resilience consistently and unidirectionally positively predicted subsequent academic engagement; and (4) The longitudinal mediating effect of psychological resilience on the prediction of academic engagement by anxiety exhibits stage-specific differentiation: in the early stage, there is a fully mediating inhibitory effect; in the later stage of school adaptation, the mediating chain disappears, leaving only the direct negative prediction of anxiety on academic engagement, with no longer any indirect effect mediated by psychological resilience. Therefore, mental health education in elementary and secondary schools should not rely on a one-size-fits-all curriculum. Instead, intervention programs should be designed in a tiered manner based on the psychological mechanisms at play during students' initial adaptation phase and their subsequent phase of stable school life, balancing emotional support with the cultivation of psychological resources to simultaneously safeguard students' mental health and foster long-term academic engagement.

## 1 Introduction

The junior secondary school years represent a crucial developmental phase during early adolescence, during which students must cope with multiple pressures, including a significant increase in academic difficulty, shifts in interpersonal relationships, and the reconstruction of self-identity. These pressures can easily lead to persistent emotional tension, excessive worry, and increased psychological burden among students, resulting in varying degrees of anxiety. Such anxiety not only has a negative impact on individual mental health but may also directly interfere with learning and affect long-term academic adjustment (Kristensen et al., 2023; Gao, 2023; Ha and Cipriano, 2025).

Among junior secondary school students, anxiety is the most common negative emotion, with a detection rate significantly higher than that of other emotional problems. The primary manifestations of anxiety include excessive preoccupation with academic performance and assessment, tension and apprehension regarding interactions with classmates, teachers, and peers, as well as persistent psychological pressure and unease in school settings such as classroom performance, examinations, and group activities. Such emotions are typically accompanied by significant physical reactions, such as headaches, stomach discomfort, and increased heart rate, and may also manifest as avoidance behaviors, such as shirking study tasks, reducing social interaction, or refusing to take on responsibilities (Dong et al., 2025). Students in a prolonged state of anxiety are prone to inattention, reduced engagement in learning, and diminished classroom participation. Furthermore, their sense of self-efficacy and self-confidence may be undermined, further exacerbating negative self-perception and hindering the acquisition of academic skills and the development of independent learning abilities. Consequently, educators, parents, and mental health professionals should attach great importance to students' anxiety and implement scientific, systematic interventions to mitigate its negative effects and reduce the cumulative damage anxiety inflicts on adolescents' psychological development and academic performance (Du et al., 2024; Ernst et al., 2023).

Psychological resilience constitutes a key psychological resource, allowing individuals to preserve adaptive functioning and promptly recover mental equilibrium in response to stressors, setbacks, or difficult circumstances. It reflects an individual's capability to remain goal-oriented, maintain emotional stability, and actively respond to challenges even in adverse circumstances (Godor et al., 2024; Marquez et al., 2023; Azpiazu Izaguirre et al., 2024). This psychological resource not only helps students cope with everyday difficulties in their studies or personal lives but also serves as a protective mechanism when they encounter emergencies or major setbacks, mitigating the effects of different negative emotions on behavior and psychological wellbeing (Li and Quan, 2025). Psychological resilience encompasses multi-level traits, such as persistence, emotional regulation, optimism, and problem-solving ability, enabling individuals to maintain a positive attitude toward learning and life even under high-pressure conditions (Wang and King, 2025).

Previous cross-sectional studies have shown that psychological resilience is significantly negatively correlated with anxiety levels: students with higher psychological resilience typically report lower levels of anxiety. At the same time, psychological resilience is positively correlated with academic engagement, suggesting that students with higher psychological resilience demonstrate greater vitality, focus, and persistence in their studies. From a classical theoretical perspective, the correlations among these variables have a solid theoretical foundation. According to Resource Conservation Theory (Hobfoll, 1989), anxiety is a depleting negative emotion that continuously depletes an individual's internal psychological resources, such as cognitive and emotional resources, whereas psychological resilience is a crucial protective psychological resource. Consequently, there is a natural inverse relationship between the two; sufficient psychological resources enable individuals to maintain a stable learning state, which also explains the positive association between psychological resilience and academic engagement. From the perspective of the stress-coping theory (Lazarus and Folkman, 1984), anxiety is a stress response generated when individuals face academic or interpersonal pressures. Psychological resilience, as a core stress-coping ability, helps individuals effectively regulate negative emotions, which is a key reason for the negative correlation between the two (Fletcher and Sarkar, 2013). However, such cross-sectional or static studies only reveal correlations among variables and fail to clarify the direction or temporal sequence of causal relationships (Sahib et al., 2025; Chen et al., 2021). This ambiguity in causality limits the scientific design of intervention strategies and makes it difficult to specifically enhance students' psychological resilience and academic performance in educational practice.

Academic engagement denotes a positive, enduring, and fulfilling psychological condition displayed by students throughout the learning process. It is reflected not only in their enthusiasm and interest in learning activities but also in their capacity to sustain attention and consistent effort during learning (Pan and Yao, 2023; Wang, 2025). This construct is generally conceptualized as encompassing three primary dimensions: vigor, dedication, and focus. Vigor refers to the energy and initiative students demonstrate in their studies; dedication reflects students' sense of responsibility and commitment to learning tasks; and focus indicates students' ability to sustain attention over extended periods during learning activities (Wang et al., 2025). Together, these three dimensions constitute key indicators for measuring the quality of academic engagement and intrinsic motivation, serving as central references for understanding students' learning behaviors and psychological states.

Existing research evidence suggests that negative emotions, such as anxiety, stress, or depression, can substantially diminish students' engagement in learning, leading to a lack of motivation, distracted attention, and reduced classroom participation. At the same time, positive psychological resources, particularly psychological resilience, self-efficacy, and emotional regulation, may act as buffers or mediators between negative emotions and academic engagement, helping to mitigate the negative impact of emotional stress on academic performance (Yang et al., 2024). However, these conclusions are largely derived from cross-sectional studies and lack a dynamic analysis of how these variables change over time.

Among junior secondary school students, the dynamic, cross-temporal interaction patterns between anxiety, psychological resilience, and academic engagement remain unclear. It is currently uncertain whether anxiety and psychological resilience exhibit a reciprocal predictive relationship, that is, whether anxiety inhibits the development of psychological resilience over time or whether psychological resilience can effectively alleviate anxiety. Nor is it clear whether anxiety and psychological resilience can reliably predict subsequent levels of academic engagement. More importantly, it remains to be clarified whether psychological resilience acts as a mediating variable that plays a key role in the long-term mechanism through which anxiety affects academic engagement, including whether this mediating effect exhibits stage-specific dynamic characteristics (Liu, 2024). These unresolved issues not only limit our theoretical understanding of adolescents' learning behavior but also affect the scientific design and practical application of educational interventions.

The association between anxiety and academic engagement can involve psychological resilience as either a moderator or a mediator (Lau, 2022). Moderation examines static variable interactions, testing whether trait resilience shifts the magnitude or direction of the anxiety–engagement association. This analytical approach requires no clear temporal ordering of variables and is typical for cross-sectional research. While this moderating framework appears partially compatible with Conservation of Resources (COR) theory—one may assume resilience buffers how strongly anxiety undermines student engagement—it merely captures a static conditional relationship, failing to reflect Hobfoll (2001) core resource loss spiral premise of COR theory.

Given our three-wave longitudinal design intended to unpack time-varying relational pathways, we specified an anxiety → psychological resilience → academic engagement mediation structure. Per COR theory, sustained anxiety consumes people's psychological resources and reshapes resilience, a vital personal resource; shifts in resilience subsequently transmit anxiety's downstream effects on academic engagement. This sequential cause-mediator-outcome pathway fits the analytical logic of longitudinal mediation (Hobfoll, 2001), which adequately captures dynamic, staged variable transmission and matches our original research design. Practically, this mediation structure pinpoints resilience as a targeted intervention entry, offering actionable references for school mental health services and academic tutoring. Balancing research aims, theoretical alignment with COR theory and applied value, we adopted the longitudinal mediation model. We nevertheless recognize the theoretical plausibility of the moderating framework and note it as a viable avenue for follow-up exploration.

In summary, building on the theoretical framework and identified research gaps, the present study adopted a three-wave longitudinal design with assessments spaced 3 months apart. Using cross-lagged models and longitudinal mediation analyses, this investigation aims to examine the temporal dynamics and underlying mechanisms connecting these three constructs. Specifically, the study investigates the following temporal relationships: (1) psychological resilience and anxiety; (2) anxiety and academic engagement; (3) psychological resilience and academic engagement; and (4) the potential mediating function of psychological resilience in the pathway from anxiety to academic engagement among junior high school students. These analyses aim to provide empirically grounded insights for the design of mental health interventions and academic support strategies within the junior secondary school context.

## 2 Materials and methods

### 2.1 Study participants

This study selected seventh- and eighth-grade students from a full-time general junior high school in eastern China as the research subjects, including 326 seventh-graders and 284 eighth-graders; 312 boys and 298 girls; with an average age of 13.26 years. This study did not include ninth-grade students, primarily for three reasons. Ninth-grade students face the heavy workload of high school entrance exams; administering multiple questionnaires could disrupt normal teaching schedules and, to some extent, violate research ethics; Chinese ninth-grade students are in the graduation transition period, at this stage, students are required to make a choice between regular senior high schools and vocational secondary schools. At this stage, students exhibit significant mobility—such as transferring schools, preparing for exams in different locations, or changing after-school tutoring institutions—which would substantially increase the risk of sample attrition in longitudinal tracking and make it difficult to ensure data integrity. This study focuses on the dynamic development of early adolescent emotions and academic behaviors. Grades seven and eight are critical stages during which psychological traits and learning states tend to stabilize, whereas Grade nine students are primarily focused on exam preparation, which deviates from the observational focus of this study.

Cluster sampling was used to implement three consecutive rounds of longitudinal assessment. During data sorting and screening, we made a clear distinction between participants submitting careless answers and those lost to follow-up. Specifically, attrition was defined as participants who completed the T1 baseline survey but did not take part in either the T2 or T3 follow-up assessments. Questionnaires containing random or inconsistent responses were flagged and excluded using embedded attention-check items. At baseline (T1), surveys were administered to 750 seventh- and eighth-grade students. 18 questionnaires were discarded for problematic responding, resulting in an initial valid sample of 732 students. For the 3-month follow-up (T2), 25 of these 732 students failed to continue participation, while another 13 questionnaires were ruled invalid owing to careless completion. This left 694 usable responses at this measurement point. At the 6-month follow-up (T3), 69 participants from the T2 sample dropped out of the study, and an additional 15 questionnaires were excluded for poor response quality. The final analytic sample consisted of 610 adolescents who provided complete data across all three survey waves.

The three rounds of surveys were conducted in late September 2025, late December 2025, and late March 2026, respectively. Due to the scheduling constraints of Chinese primary and secondary schools, the three-wave longitudinal design—spaced 3 months apart—could not be completed within a single semester; consequently, the study period naturally spanned both the fall and spring semesters and included the winter break. This study deliberately avoided the periods at the beginning and end of the semester, when student states are subject to significant fluctuations, and instead chose to administer the assessments midway through the semester to minimize interference with the observed data from factors such as school adjustment and pre-exam psychological fluctuations (Wei et al., 2025). Furthermore, compared to the long summer vacation, the winter break is shorter, and changes in students' psychological states and learning rhythms are less pronounced, which can further reduce the potential impact of vacation length on the core variables (Cooper et al., 1996).

### 2.2 Research instruments

#### 2.2.1 Screening questionnaire for anxiety disorders in children (SCARED)

The Chinese translation of the Screening Questionnaire for Anxiety Disorders in Children was used to survey Chinese secondary school students. The questionnaire comprises 41 items covering five dimensions: somatisation, generalized anxiety, separation anxiety, school phobia, and social phobia (Birmaher et al., 1997). Sample items include “I often worry whether others like me” and “I always feel nervous and restless.” The scale uses a zero to two scoring system, ranging from zero (“No such problem”) to two (“Often”). The total score ranges from 0 to 82, with higher scores reflecting greater severity of anxiety. Prior research has demonstrated that the Chinese version of the SCARED is a reliable instrument for evaluating anxiety in children (Su et al., 2008). Internal consistency was good across the three measurement time points in this study. Cronbach's α coefficients, rounded to three decimal places, were 0.939 (T1), 0.950 (T2), and 0.946 (T3). McDonald's ω coefficients were largely consistent with these, at 0.939 (T1), 0.950 (T2), and 0.946 (T3), indicating that the scales demonstrated high and stable reliability at all three time points.

#### 2.2.2 Connor-davidson resilience scale (CD-RISC)

The Chinese translation of the Connor-Davidson Resilience Scale was used to survey Chinese junior high school students. The scale comprises 25 items covering three dimensions: resilience, strength, and optimism (Connor and Davidson, 2003). Sample items include “I can achieve my goals” and “I do not get discouraged by failures.” The scale is scored on a zero to four scale, ranging from zero (“never”) to four (“always”), with a total score ranging from 0 to 100; a higher score indicates a higher level of psychological resilience. Previous research has also demonstrated that the Chinese version of the CD-RISC possesses sound psychometric properties among secondary school students (Yu and Zhang, 2007). Internal consistency was good across the three measurement time points in this study. Cronbach's α coefficients, rounded to three decimal places, were 0.941 (T1), 0.942 (T2), and 0.939 (T3). McDonald's ω coefficients were largely consistent with these, at 0.941 (T1), 0.943 (T2), and 0.940 (T3), indicating that the scales demonstrated high and stable reliability at all three time points.

#### 2.2.3 University of washington engagement scale (UWES-S)

The Chinese translation of the UWES-S was used to survey Chinese junior high school students. The scale consists of 17 items spanning three dimensions: vigor, dedication, and focus (Schaufeli et al., 2002). Sample items include “I find learning challenging” and “I am proud of my learning.” Responses are recorded on a 0 to 6 rating scale, with total scores ranging from 0 to 102, where higher scores reflect greater levels of academic engagement. Previous studies have confirmed that the Chinese version of the UWES-S demonstrates adequate reliability among secondary school students (Teuber et al., 2021). Internal consistency was good across the three measurement time points in this study. Cronbach's α coefficients, rounded to three decimal places, were 0.905 (T1), 0.907 (T2), and 0.895 (T3). McDonald's ω coefficients were largely consistent with these, at 0.905 (T1), 0.907 (T2), and 0.895 (T3), indicating that the scales demonstrated high and stable reliability at all three time points.

### 2.3 Administration procedure

This study employed a class-based group administration method, with a standardized set of instructions read aloud by an examiner who had received standardized training. During the administration, participants completed the questionnaire independently and anonymously; no personally identifiable information was collected. Only non-invasive, non-medical psychological questionnaire surveys were conducted; no invasive medical procedures, such as blood draws or physiological tests, were performed. All raw data were stored on encrypted devices, kept strictly confidential, never disclosed externally, and used solely for the statistical analysis of this study. Data from the three follow-up rounds were matched using pre-assigned unique identifiers to ensure the integrity and accuracy of the longitudinal data. All research subjects together with their legal guardians needed to carefully go through the informed consent paperwork prior to taking part in the evaluation procedures, thereby fully safeguarding the participants' right to informed consent and their rights and interests.

### 2.4 Statistical methods

Descriptive statistics, correlation analyses, and reliability assessments were conducted in SPSS, whereas AMOS was used to build cross-lagged and longitudinal mediation models. This study employs a cross-lagged panel model (CLPM) based on observed variables for data analysis. This model features a straightforward computational process and efficient parameter estimation; it is well-suited to the sample size and data structure of this three-wave longitudinal study, clearly reveals the longitudinal predictive relationships among variables, and offers strong practicality and interpretability of results. Model fit was assessed using indices such as χ^2^/df, GFI, AGFI, CFI, RMSEA, and SRMR. According to the thresholds recommended by Hu and Bentler, acceptable model fit was specified as χ^2^/df < 3, GFI > 0.90, AGFI > 0.90, CFI > 0.90, RMSEA < 0.08, and SRMR < 0.08. The mediation analysis was conducted using the PROCESS macro (version 4.2) in SPSS (Department of Psychology, The Ohio State University, Columbus, OH, United States), and all statistical tests applied a two-tailed significance level of *p* < 0.05 (Hu and Bentler, 1999).

## 3 Results

### 3.1 Attrition analysis

To examine whether sample attrition during the three rounds of longitudinal follow-up introduced systematic bias, During the data cleaning phase of this study, in addition to excluding samples that did not complete all three rounds of testing, we also implemented multi-level screening and exclusion of careless response samples using methods such as response duration filtering and the identification of consistent response patterns (Curran, 2016; Ward and Meade, 2023), this study employed an independent-samples *t*-test to compare the baseline scores of the core variables at the first measurement point (T1) between participants who completed all three assessments (completion group, *n* = 610) and those who dropped out midway (attrition group, *n* = 140). The corresponding results are presented in Table 1.

**Table 1:** Descriptive statistics and independent-samples *t*-test results for ANX, PR, and AE between completers and dropouts.

Variable	Completers (*n* = 610) M ±SD	Dropouts (*n* = 140) M ±SD	*t*	df	*p*	Cohen's d	95% CI for *d*
ANX	41.92 ± 18.02	41.95 ± 17.92	−0.015	748	0.988	−0.001	[−0.185, 0.182]
PR	50.70 ± 22.81	52.44 ± 22.90	−0.813	748	0.416	−0.076	[−0.260, 0.108]
AE	51.40 ± 21.68	49.64 ± 22.39	0.860	748	0.39	0.081	[−0.103, 0.264]

ANX, anxiety; PR, psychological resilience; AE, academic engagement; M, mean.

As shown in Table 1, for the three core variables, anxiety, psychological resilience, and academic engagement, no statistically significant differences were observed between the completion group and the attrition group in their baseline scores (anxiety: *t* (748) = −0.015, *p* = 0.988; psychological resilience: *t* (748) = −0.813, *p* = 0.416; academic engagement: *t* (748) = 0.860, *p* = 0.390). The effect sizes for all three comparisons were extremely small (|Cohen's *d*| < 0.10), and the 95% confidence intervals for all effect sizes included zero, indicating no substantial differences.

These results indicate that there were no systematic differences in the initial levels of the core variables between participants who completed the study and those who withdrew during the study. This suggests that sample attrition was random and did not introduce significant selection bias into the final analysis sample. Consequently, the final sample in this study can be regarded as an unbiased representation of the initial cohort in terms of the core variables, providing a reliable basis for the analysis of the longitudinal follow-up data (Steinhausen et al., 2020).

### 3.2 Common method bias test

To assess the potential for common method bias, Harman's single-factor test was conducted, and make a comprehensive assessment based on relevant methodological recommendations (Podsakoff et al., 2003). The results indicated that the first unrotated factor accounted for 19.47% of the total variance, which is well below the 40% threshold, suggesting that common method bias is not a serious concern in this study.

### 3.3 Correlations between psychological variables across the three time points

The Pearson correlation coefficients for each variable across the three measurements are presented in Table 2. The results indicate that all psychological variables are significantly correlated with one another, and the direction of these correlations is consistent with theoretical expectations.

**Table 2:** Descriptive statistics and intercorrelations among ANX, PR, and AE across three time points.

Variables	1.	2.	3.	4.	5.	6.	7.	8.	9.
1. ANX (T1)	1								
2. ANX (T2)	0.622^**^	1							
3. ANX (T3)	0.628^**^	0.722^**^	1						
4. PR (T1)	−0.466^**^	−0.451^**^	−0.508^**^	1					
5. PR (T2)	−0.488^**^	−0.610^**^	−0.612^**^	0.697^**^	1				
6. PR (T3)	−0.340^**^	−0.396^**^	−0.541^**^	0.564^**^	0.609^**^	1			
7. AE (T1)	−0.391^**^	−0.259^**^	−0.277^**^	0.305^**^	0.252^**^	0.228^**^	1		
8. AE (T2)	−0.269^**^	−0.184^**^	−0.261^**^	0.309^**^	0.534^**^	0.321^**^	0.390^**^	1	
9. AE (T3)	−0.321^**^	−0.357^**^	−0.305^**^	0.380^**^	0.482^**^	0.481^**^	0.350^**^	0.477^**^	1
M	41.92	42.72	46.04	50.70	53.73	58.63	51.40	50.01	48.72
SD	18.02	19.42	18.66	22.81	22.95	22.23	21.68	21.58	20.80

ANX, anxiety; PR, psychological resilience; AE, academic engagement; M, mean; T1, time point 1; T2, time point 2; T3, time point 3; *N* = 610; ^**^p < 0.01, two-tailed.

This study conducted Pearson correlation analyses on anxiety, psychological resilience, and academic engagement across the three measurement points. The results indicate that all variables are significantly correlated with one another, and the direction of these correlations is consistent with theoretical assumptions. The specific analysis is as follows:

Table 2 displays the Pearson correlation coefficients of anxiety, psychological resilience, and academic engagement among junior high school students across the three measurement occasions. As shown in the table, significant correlations exist among all psychological variables (*p* < 0.01), and the direction of these correlations aligns with theoretical assumptions. This indicates that anxiety, psychological resilience, and academic engagement are statistically interrelated, providing a foundation for further exploration of their dynamic relationships (Liu et al., 2025).

Among junior high school students, anxiety demonstrated a significant negative association with psychological resilience, with correlation coefficients ranging from −0.612 to −0.340. This suggests that anxiety can undermine students' concentration and engagement in learning. Conversely, psychological resilience demonstrated a significant positive association with academic engagement, with correlation coefficients ranging from 0.228 to 0.534. This suggests that students with higher psychological resilience are able to maintain stronger academic motivation and task focus, confirming that psychological resilience is a key protective psychological resource for buffering academic stress and enhancing academic engagement.

Taken together, these findings not only validate theoretical expectations but also provide an empirical foundation for subsequent longitudinal analyses and the construction of mediation models, offering preliminary evidence for elucidating the time-series relationships and underlying mechanisms among anxiety, psychological resilience, and academic engagement.

### 3.4 Measurement invariance across three time points

To ensure that the three waves of longitudinal tracking data could be used for comparisons of latent variable means and cross-lagged model analyses, this study employed multi-group confirmatory factor analysis (MGCFA) to sequentially test the construct invariance, metric invariance, and scalar invariance of the anxiety, psychological resilience, and academic engagement scales at the three measurement time points: T1, T2, and T3. This study sequentially constructed three nested models to conduct measurement invariance tests: structural invariance, metric invariance, and scalar invariance. The criteria for assessing overall model fit were: a chi-square-to-degrees-of-freedom ratio (χ^2^/df) < 3, the root mean square error of approximation (RMSEA) is < 0.08, the comparative fit index (CFI) is >0.90, the Tucker–Lewis index (TLI) is >0.90, and the standardized root mean square residual (SRMR) is < 0.08 (Hu and Bentler, 1999). The criterion for comparing nested models is as follows: if ΔCFI ≤ −0.010 and ΔRMSEA ≤ 0.015, measurement invariance is considered to hold (Chen, 2007). The fit results for measurement invariance are presented in Table 3.

**Table 3:** Fit indices for configural, metric, and scalar measurement invariance across variables.

Variable	Model type	χ^2^/df	RMSEA	CFI	TLI	SRMR	ΔCFI	ΔRMSEA
ANX	Configural invariance	1.133	0.015	0.986	0.985	0.028	—	—
	Metric invariance	1.146	0.015	0.984	0.983	0.035	−0.002	0
	Scalar invariance	1.14	0.015	0.984	0.984	0.035	0	0
PR	Configural invariance	1.197	0.018	0.991	0.99	0.024	—	—
	Metric invariance	1.307	0.022	0.985	0.985	0.039	−0.006	0.004
	Scalar invariance	1.312	0.023	0.984	0.984	0.039	−0.001	0.001
AE	Configural invariance	1.067	0.01	0.997	0.997	0.023	—	—
	Metric invariance	1.18	0.017	0.993	0.992	0.035	−0.004	0.007
	Scalar invariance	1.152	0.016	0.993	0.993	0.036	0	−0.001

As shown in Table 3, the overall fit of the models at all levels for anxiety, resilience, and academic engagement is good. The χ^2^/df values for each model range from 1.067 to 1.312, all of which are less than the critical value of 3; the RMSEA values fall within the range of 0.010 to 0.023, which is < 0.08; CFI and TLI were both above 0.98, far exceeding the 0.90 cutoff threshold; SRMR ranged from 0.023 to 0.039, which is < 0.08, indicating ideal model fit.

#### 3.4.1 Structural invariance

The model fit indices for the three-variable structural models all met the criteria, indicating that the factor structures of anxiety, psychological resilience, and academic engagement were completely consistent across the three measurement time points. The pattern of item-to-latent variable mappings did not change over time; thus, structural invariance holds, providing a foundation for subsequent constraint tests.

#### 3.4.2 Measurement invariance

After constraining the factor loadings to be equal across time, the corresponding ΔCFI values for anxiety, psychological resilience, and academic engagement were −0.002, −0.006, and −0.004, respectively, all meeting the criterion of ΔCFI ≥ −0.01. Measurement invariance was established, indicating that the magnitude of variation in the latent variables corresponds to the magnitude of variation in observed item scores across the three measurement waves, allowing for cross-temporal comparisons of latent variable variances and correlation coefficients.

#### 3.4.3 Scalar invariance

Building on the equivalence of loadings, we further constrained the item intercepts to be equal across time. Compared to the metric model, the ΔCFI values for anxiety, psychological resilience, and academic engagement were 0.000, −0.001, and 0.000, respectively; none exceeded the acceptable range of decline, and scalar invariance was established. These results indicate that when the means of the latent variables are the same, there is no systematic temporal shift in the mean scores of the observed items, thereby satisfying the prerequisite for cross-temporal comparisons of latent variable means.

In summary, anxiety, psychological resilience, and academic engagement all passed the configural, metric, and scalar invariance tests across the three measurement waves. The scales demonstrate good cross-temporal measurement equivalence, allowing for further analysis of longitudinal differences in latent variable means and the development of cross-lagged models.

### 3.5 Temporal relationship between anxiety and psychological resilience

**Figure 1:**
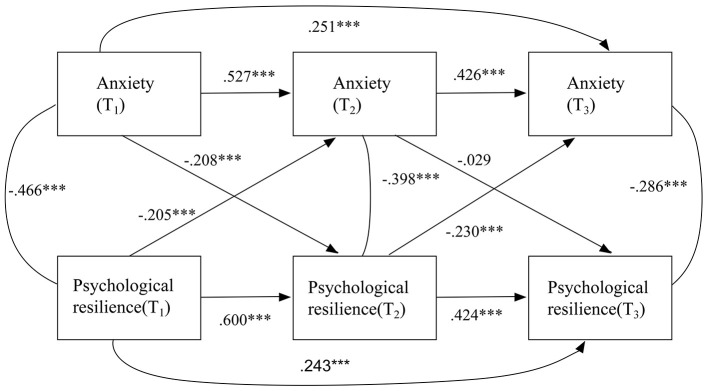
Three-wave cross-lagged model of anxiety and psychological resilience with standardized path coefficients. ***p < 0.001, two-tailed.

A three-wave cross-lagged panel model was implemented to examine the temporal association between psychological resilience and anxiety, as illustrated in Figure 1. After standardization, all autoregressive paths and cross-lagged paths except the predictive path from T2 psychological resilience to T3 anxiety were statistically significant. The model demonstrated satisfactory fit, with the following fit indices: χ^2^/df = 2.443, *p* = 0.087, GFI = 0.997, AGFI = 0.972, CFI = 0.999, and RMSEA = 0.049, all of which met the criteria for acceptable model fit.

The present study used a cross-lagged model to investigate the longitudinal association between anxiety and psychological resilience. The results revealed a notable bidirectional negative predictive relationship over time: anxiety at T1 negatively and significantly predicted psychological resilience at T2 (β = −0.208, *p* < 0.001), whereas psychological resilience at T1 also exerted a significant negative predictive effect on anxiety at T2 (β = −0.205, *p* < 0.001). This suggests that elevated levels of anxiety in the early stage significantly undermine subsequent psychological resilience, while higher initial psychological resilience effectively alleviates anxiety in the later stage, forming a mutually reinforcing negative cycle between the two in the early phase of the study.

During the T2 to T3 period, the protective effect of psychological resilience on anxiety persisted: psychological resilience at T2 significantly and negatively predicted anxiety at T3 (β = −0.230, *p* < 0.001), whereas the predictive path from T2 anxiety to T3 psychological resilience was no longer significant. This indicates that, over time, the negative impact of early anxiety on subsequent psychological resilience gradually diminished, leaving only the long-term buffering effect of psychological resilience on anxiety. Furthermore, both anxiety and psychological resilience demonstrated strong temporal stability. All autoregressive paths for anxiety were significant: T1 anxiety predicted T2 anxiety (β = 0.527, *p* < 0.001) and T3 anxiety (β = 0.251, *p* < 0.001), and T2 anxiety also predicted T3 anxiety (β = 0.426, *p* < 0.001). Psychological resilience similarly demonstrated stable continuity over time: T1 psychological resilience predicted T2 psychological resilience (β = 0.600, *p* < 0.001) and T3 psychological resilience (β = 0.243, *p* < 0.001), and T2 psychological resilience also predicted T3 psychological resilience (β = 0.424, *p* < 0.001).

The results of the cross-lagged model indicate that T1 anxiety and psychological resilience exhibit a significant negative bidirectional predictive relationship; longitudinal effects show a divergence, with the negative predictive effect of psychological resilience on subsequent anxiety remaining stable across time points, while the negative predictive effect of anxiety on subsequent psychological resilience gradually weakens over time.

### 3.6 Temporal relationship between anxiety and academic engagement

**Figure 2:**
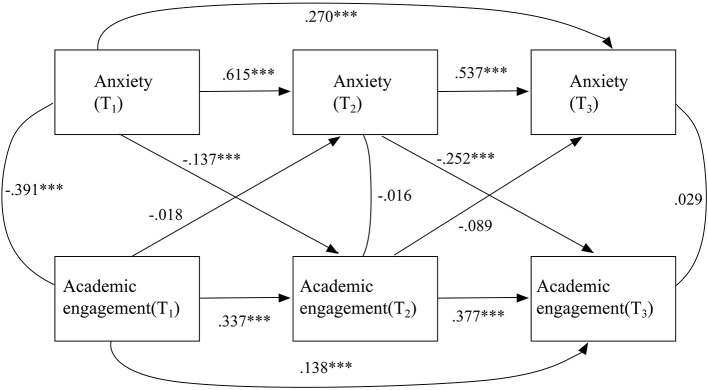
Three-wave cross-lagged model of anxiety and academic engagement with standardized path coefficients. ***p < 0.001, two-tailed.

A three-wave cross-lagged framework was applied to examine the temporal associations between academic engagement and anxiety, as illustrated in Figure 2. All autoregressive paths and cross-lagged paths predicting later academic engagement from prior anxiety yielded statistically significant standardized coefficients, whereas cross-lagged paths running from academic engagement to subsequent anxiety were not statistically significant. The model demonstrated acceptable fit, as indicated by the following indices: χ^2^/df = 0.075, *p* = 0.928, GFI = 1.000, AGFI = 0.999, CFI = 1.000, and RMSEA = 0.000.

The results of the model revealed a unidirectional, asymmetric longitudinal relationship between anxiety and academic engagement.

A stable negative predictive effect of T1 anxiety on T2 academic engagement was observed (β = −0.137, *p* < 0.001). From T2 to T3, the negative predictive effect of T2 anxiety on T3 academic engagement persisted and was further strengthened (β = −0.252, *p* < 0.001). This indicates that higher levels of anxiety at an earlier stage are associated with lower levels of subsequent academic engagement, and that this negative effect persists over time.

The reverse predictive path from academic engagement to anxiety was not significant. No significant cross-temporal predictive path from academic engagement to anxiety was found in the model, meaning that T1 academic engagement did not predict T2 anxiety, nor was there a long-term effect of T1 academic engagement on T3 anxiety. This indicates that increased academic engagement cannot effectively alleviate subsequent anxiety, and there is no bidirectional cyclical relationship between the two.

Both anxiety and academic engagement exhibited strong autoregressive effects. For anxiety, T1 anxiety predicted T2 anxiety (β = 0.615, *p* < 0.001) and T3 anxiety (β = 0.270, *p* < 0.001), while T2 anxiety predicted T3 anxiety (β = 0.537, *p* < 0.001), indicating that anxiety exhibits significant temporal continuity. For academic engagement, T1 academic engagement predicted T2 academic engagement (β = 0.337, *p* < 0.001) and T3 academic engagement (β = 0.138, *p* < 0.001), while T2 academic engagement predicted T3 academic engagement (β = 0.377, *p* < 0.001), indicating that academic engagement also exhibits stable cross-temporal characteristics.

Cross-lagged analysis results indicate that anxiety consistently and negatively predicts subsequent academic engagement, and this inhibitory effect gradually strengthens over time; high initial anxiety persistently weakens students' long-term concentration and engagement in learning. The model did not detect a significant reverse predictive path from academic engagement to anxiety, indicating that academic engagement is unlikely to buffer or alleviate anxiety in adolescents. The longitudinal relationship between the two exhibits distinct asymmetric and unidirectional characteristics, with anxiety serving as a core risk factor influencing academic engagement, and its negative effects demonstrating cumulative and persistent nature. This temporal pattern suggests that school-based psychological interventions should prioritize addressing student anxiety in order to achieve long-term improvements in adolescents' academic engagement.

### 3.7 Temporal relationship between psychological resilience and academic engagement

**Figure 3:**
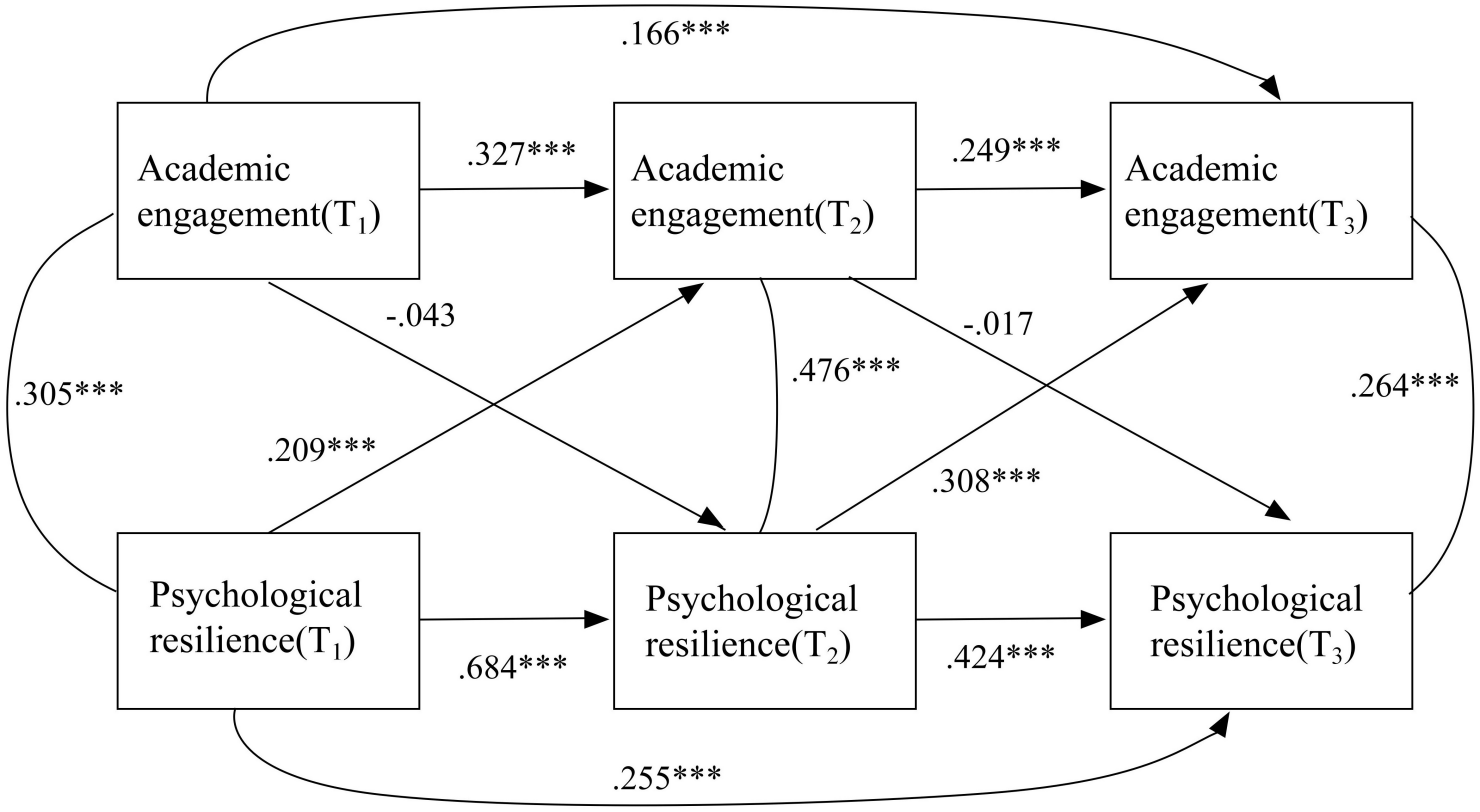
Three-wave cross-lagged model of psychological resilience and academic engagement with standardized path coefficients. ***p < 0.001, two-tailed.

To examine the temporal associations between psychological resilience and academic engagement, a three-wave cross-lagged panel model was constructed, as illustrated in Figure 3. All autoregressive paths and cross-lagged paths from psychological resilience to subsequent academic engagement yielded statistically significant standardized coefficients, while cross-lagged paths running from academic engagement to later psychological resilience were non-significant. The fit indices for the model were as follows: χ^2^/df = 2.136, *p* = 0.118, GFI = 0.998, AGFI = 0.976, CFI = 0.998, and RMSEA = 0.043. These indices satisfied the standards for acceptable model fit.

The findings show that the cross-temporal association between the two variables follows a stable, unidirectional enhancing pattern. From T1 to T2, psychological resilience had a significant positive predictive effect on subsequent academic engagement, with T1 psychological resilience significantly predicting T2 academic engagement (β = 0.209, *p* < 0.001). In contrast, the predictive pathway from T1 academic engagement to T2 psychological resilience was not statistically significant.

From T2 to T3, the positive predictive effect of psychological resilience on academic engagement persisted and strengthened: psychological resilience at T2 significantly and positively predicted academic engagement at T3 (β = 0.308, *p* < 0.001). Conversely, the predictive path from academic engagement at T2 to psychological resilience at T3 was not significant.

In addition, both psychological resilience and academic engagement exhibited strong temporal stability. All autoregressive paths for academic engagement were significant: T1 academic engagement positively predicted T2 (β = 0.327, *p* < 0.001) and T3 scores (β = 0.166, *p* < 0.001), and T2 academic engagement also positively predicted T3 (β = 0.249, *p* < 0.001). Similarly, the autoregressive paths for psychological resilience were significant, with T1 psychological resilience predicting T2 (β = 0.684, *p* < 0.001) and T3 scores (β = 0.255, *p* < 0.001), and T2 psychological resilience also positively predicting T3 (β = 0.424, *p* < 0.001).

Results from the cross-lagged model indicate that psychological resilience consistently and positively predicts subsequent academic engagement, and this promotional effect continues to strengthen over time. Students with high psychological resilience early on are able to maintain stronger academic focus and the ability to cope with challenges over the long term, thereby continuously improving their level of academic engagement. The analysis did not detect a significant reverse longitudinal predictive path from academic engagement to psychological resilience. This suggests that increased academic engagement is unlikely to shape or enhance an individual's psychological resilience, indicating a clear unidirectional relationship between the two over time. Time-series data confirm that psychological resilience is a core protective psychological resource underpinning adolescents' academic engagement, and its positive effects exhibit long-term cumulative characteristics. Based on this asymmetric relationship, school-based interventions should prioritize the cultivation of students' psychological resilience as a core strategy. Effectively enhancing psychological resilience can continuously optimize junior high school students' learning status and academic adaptation throughout the entire junior high school period.

### 3.8 Mediating role of psychological resilience between anxiety and academic engagement

The specification of the longitudinal mediation model draws on recommendations from existing research on time-separated mediation analysis, which involves separating the independent variable, the mediating variable, and the outcome variable at different time points to enhance the validity of causal inferences (Jose, 2016; Caemmerer et al., 2024).

**Figure 4:**
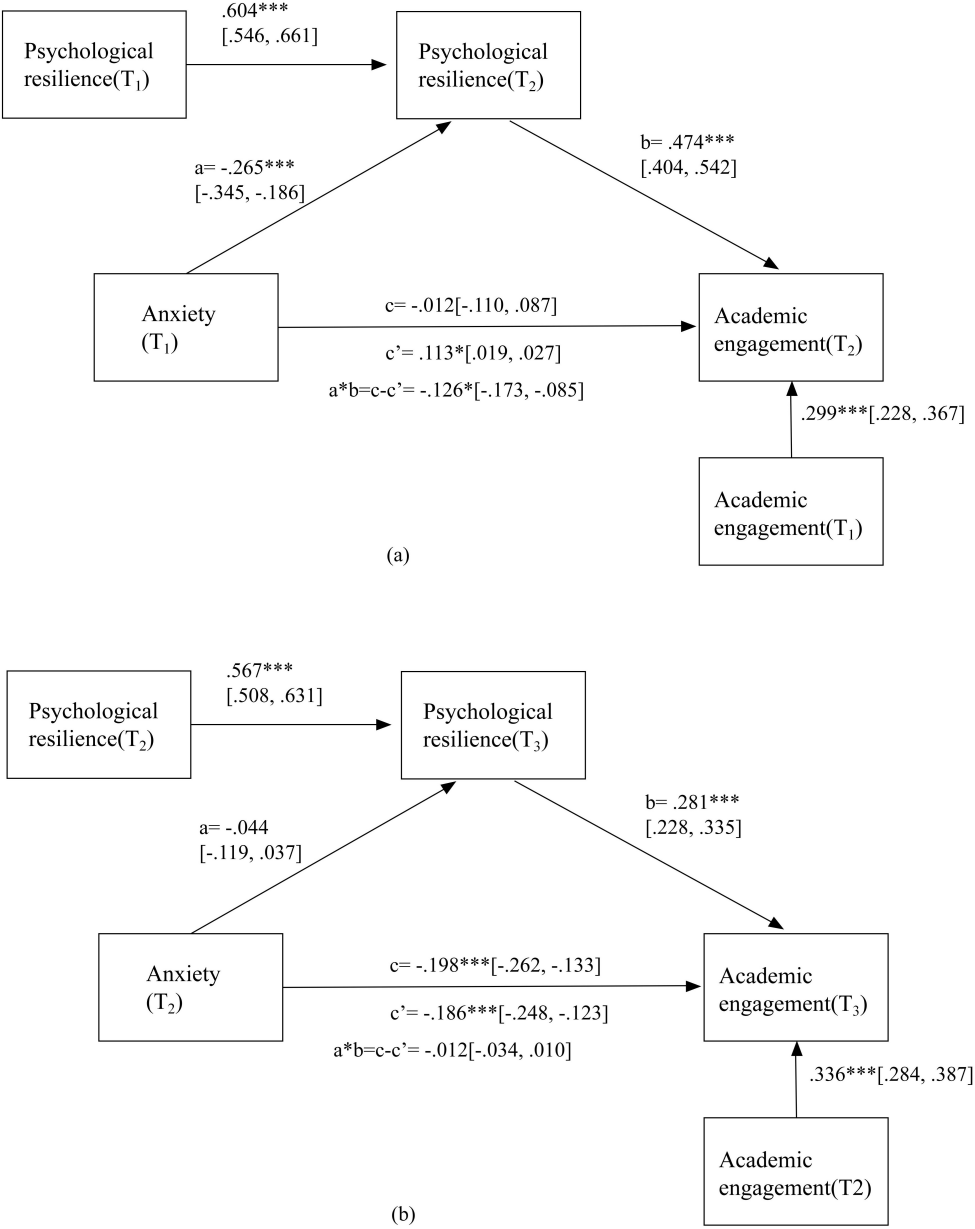
Longitudinal mediation models exploring the mediating role of psychological resilience in the relationship between anxiety and academic engagement. **(a)** Mediation model from T1 to T2. **(b)** Mediation model from T2 to T3. Path coefficients are unstandardized regression coefficients with 95% confidence intervals; ***p < 0.001, *p < 0.05, two-tailed.

To investigate the role of psychological resilience as a mediator linking anxiety to academic engagement in junior high school students, we constructed two longitudinal mediation models, as illustrated in Figure 4. Although traditional longitudinal mediation frameworks often adopt a three-wave sequential mediation framework (T1 predictor → T2 mediator → T3 outcome with baseline variables controlled), the present study adopts a two-wave lagged mediation model based on integrated theoretical and methodological rationales. We fully recognize that this three-wave sequential approach delivers robust estimates for long-term delayed indirect effects, yet its analytical setup fails to align with the core research objectives of this investigation. First, the primary aim of this work is to unpack short-term proximal lagged associations: we aim to capture how baseline anxiety concurrently predicts shifts in psychological resilience and academic engagement within a single follow-up window, rather than estimating a prolonged three-stage indirect pathway across all three measurement time points. The two-wave lagged structure enables us to capture synchronous changes in both the mediator and outcome variable triggered by baseline anxiety over one complete tracking interval, which aligns closely with our theoretical propositions concerning stage-dependent short-term mediating mechanisms. Second, the full analytical strategy of this study rests on three-wave cross-lagged panel models to unpack reciprocal longitudinal associations across anxiety, psychological resilience, and academic engagement. All cross-lagged estimations follow a consistent two-wave time lag (T1 → T2, T2 → T3). Implementing two-wave lagged mediation (T1 anxiety predicting T2 psychological resilience and T2 academic engagement) ensures methodological consistency across all statistical analyses reported herein. Introducing a separate T1 anxiety → T2 psychological resilience → T3 academic engagement sequential mediation model would create mismatched lag intervals relative to the cross-lagged panel models. Such structural inconsistency would render direct comparisons of longitudinal path coefficients unreliable and fragment the unified analytical logic running through the manuscript. During the model estimation process, the covariance among anxiety, psychological resilience, and academic engagement at the baseline time point was included to control for concurrent correlations among the variables and potential common-method effects; however, to simplify the presentation of the model, it is not shown in the figure. Model parameters were estimated using the SPSS PROCESS 4.2 plugin, and the significance of the mediating effects was tested using 5,000 Bootstrap samples.

As shown in Figure 4a, in the longitudinal mediation model between T1 and T2, psychological resilience significantly mediated the relationship between anxiety and academic engagement. Specifically, anxiety at T1 significantly and negatively predicted psychological resilience at T2 (*a* = −0.265, *p* < 0.001, 95% CI [−0.345, −0.186]), indicating that higher initial levels of anxiety significantly undermine the subsequent development of psychological resilience. At the same time, psychological resilience at T1 had a significant positive predictive effect on psychological resilience at T2 (*b* = 0.604, *p* < 0.001, 95% CI [0.546, 0.661]).

Regarding the outcome variables, psychological resilience at T2 significantly and positively predicted academic engagement at T2 (*b* = 0.474, *p* < 0.001, 95% CI [0.404, 0.542]). Furthermore, academic engagement at T1 had a significant positive predictive effect on academic engagement at T2 (*b* = 0.299, *p* < 0.001, 95% CI [0.228, 0.367]).

After controlling for mediating variables, the direct effect of anxiety at T1 on academic engagement at T2 remained a small but significant positive effect (*c*′ = 0.113, *p* < 0.05, 95% CI [0.019, 0.207]), while its overall effect was not significant (*c* = −0.012, 95% CI [−0.110, 0.087]). Bootstrap test results indicated that the indirect effect was significant (*a* × *b* = −0.126, 95% CI [−0.173, −0.085]), suggesting that psychological resilience played a significant mediating role between anxiety and academic engagement.

This study found that the total effect of T1 anxiety on T2 academic engagement was not significant, whereas its indirect effect was significant and negative. This pattern of results may reflect an inconsistent mediation or an inhibitory effect structure. Specifically, anxiety exerts a negative indirect effect on academic engagement by weakening psychological resilience, while its direct path to academic engagement exhibits a weaker positive effect; these two effects offset each other in the overall effect, resulting in a non-significant total effect.

As shown in Figure 4b, in the longitudinal mediation model between T2 and T3, the predictive effect of T2 anxiety on T3 psychological resilience was not significant (*a* = −0.044, 95% CI [−0.119, 0.037]), indicating that the influence of anxiety on psychological resilience during this time window did not reach statistical significance. At the same time, psychological resilience at T3 had a significant positive predictive effect on academic engagement at T3 (*b* = 0.281, *p* < 0.001, 95% CI [0.228, 0.335]).

Regarding the outcome variable, T2 anxiety had a significant negative predictive effect on T3 academic engagement (*c* = −0.198, *p* < 0.001, 95% CI [−0.262, −0.133]). After controlling for the mediating variable psychological resilience, the direct effect remained significant (c' = −0.186, *p* < 0.001, 95% CI [−0.248, −0.123]). However, Bootstrap test results showed that the indirect effect was not significant (*a* × *b* = −0.012, 95% CI [−0.034, 0.010]), indicating that psychological resilience did not play a significant mediating role between anxiety and academic engagement from T2 to T3.

Furthermore, the model also revealed significant time-stability effects: psychological resilience exhibited strong stability over time (*b* = 0.567, *p* < 0.001), and academic engagement also demonstrated significant stability (*b* = 0.336, *p* < 0.001), indicating that individuals follow relatively stable developmental trajectories for these key psychological and behavioral variables.

Overall, during the T2 → T3 time interval, the effect of anxiety on academic engagement was primarily manifested as a direct effect, while the mediating role of psychological resilience was not supported. A possible explanation for this result is that, as a relatively stable psychological resource, changes in psychological resilience typically require a prolonged cumulative process; therefore, within a shorter time interval, the impact of anxiety on it may not yet have fully manifested. Furthermore, psychological resilience in this study exhibited strong temporal stability, and this stability may have further weakened the additional explanatory power of the anxiety variable. At the same time, during the developmental stage of junior high school students, psychological resilience may have gradually stabilized, reducing its sensitivity to short-term situational stress.

The results indicate that the role of psychological resilience in academic engagement varies across different time periods. During the T1 → T2 phase, psychological resilience played a significant mediating role between anxiety and academic engagement, suggesting that it partially explains the mechanism through which anxiety influences academic engagement in the early stages. However, during the T2 → T3 phase, this mediating effect was not statistically significant, suggesting that its role may be influenced by the time window and individual developmental stability.

Overall, psychological resilience manifests more as a stage-specific psychological resource in the academic development of adolescents rather than as a consistent mediating factor across all time periods. These findings suggest that its mechanism of action may be jointly influenced by developmental stage, time intervals, and individual psychological stability.

## 4 Discussion

This study employs a three-wave longitudinal design combined with cross-lagged analysis to systematically investigate the temporal predictive relationships among anxiety, psychological resilience, and academic engagement among junior high school students, distinguishing itself from single-time-point cross-sectional studies that can only capture instantaneous associations (Chen et al., 2024). Numerous previous cross-sectional studies have only been able to confirm static correlations among variables; they cannot clarify the sequence of variable occurrence or long-term developmental trajectories, nor can they support the design of phased school-based intervention programs. In contrast, the three rounds of longitudinal data in this study enable the characterization of predictive chains reflecting dynamic changes in variables over time, while also testing the longitudinal mediating effect of psychological resilience, thereby addressing the shortcoming of cross-sectional studies regarding the lack of temporal evidence. However, this study still has inherent design limitations: relying solely on correlational longitudinal data makes it impossible to completely eliminate the interference of unobserved confounding variables. All analytical results can only describe temporal patterns and cannot establish deterministic causality. Based on the longitudinal data, this study can only make a limited inference: psychological resilience serves as a stable, protective predictor at the emotional and academic levels, providing a reference for the design of phased psychological development and academic support programs in schools.

### 4.1 The bidirectional temporal relationship between anxiety and psychological resilience

Longitudinal data from this study indicate that early anxiety and psychological resilience during the early junior high school years exhibit a significant bidirectional negative temporal association: higher levels of anxiety at the outset negatively predict relatively lower levels of psychological resilience later on, while students with more abundant initial psychological resilience resources tend to maintain lower levels of anxiety over time. This overall pattern aligns with the core logic of stress coping theory and is generally consistent with the findings of most longitudinal studies on adolescents (Jianping et al., 2024; Llistosella et al., 2024; Zhang et al., 2022). From a psychological mechanism perspective, persistent anxiety continuously depletes an individual's limited cognitive and emotional regulation resources, weakening their capacity for psychological recovery and environmental adaptation, which in turn hinders the sustained development of psychological resilience resources. Conversely, students with higher levels of psychological resilience possess more mature stress coping and emotional regulation strategies, enabling them to better buffer against negative external stimuli and, to a certain extent, anticipate lower levels of anxiety accumulation.

Considering the physical and psychological developmental characteristics of junior high school students, at the beginning of a new school year, new students' emotional states and internal psychological resources have not yet stabilized, leading to a mutually constraining temporal relationship between the two. As school adaptation gradually takes place, individual psychological resilience tends to stabilize, and the impact of early, transient emotional fluctuations on subsequent psychological development gradually weakens, leaving only the long-term protective predictive effect of psychological resilience on anxiety. This temporal differentiation complements the long-term developmental patterns that most previous short-term, two-wave longitudinal studies have struggled to capture, further suggesting that psychological interventions in junior high school should focus on the critical adaptation period in seventh grade. By cultivating psychological resilience early on, such interventions can help students build long-term, stable psychological protective resources. It should be carefully noted that the bidirectional association observed in this study represents only a temporal predictive relationship. Due to the limitations of a non-experimental longitudinal design, the study cannot rule out the combined influence of individual traits and external environmental variables. Furthermore, this study can only reflect horizontal differences among individuals and cannot depict the dynamic changes in a single student's psychological indicators over time; therefore, one should not overinterpret the absolute causal inhibitory effects between the two variables.

### 4.2 Unidirectional negative prediction of anxiety on academic engagement

The longitudinal time-series analysis in this study shows that early levels of anxiety consistently and negatively predict an individual's subsequent academic engagement; this predictive effect gradually strengthens over the follow-up period, and no reverse predictive path from academic engagement to anxiety was observed. The overall temporal association patterns are generally consistent with the findings of longitudinal studies on the emotional and academic development of adolescents (Zhang et al., 2023; Eriksen and Bru, 2023). Compared to traditional cross-sectional studies, which are limited to capturing only static relationships between variables, this study, based on multi-wave longitudinal data, clarifies the temporal sequence of these variables and more clearly reveals the developmental pattern in which anxiety precedes academic engagement. From an internal mechanism perspective, anxiety consumes students' limited cognitive and attentional resources, easily inducing academic avoidance behaviors and negative learning motivation, causing students with high anxiety in the early stages to consistently exhibit lower levels of concentration and engagement in subsequent learning. It must be rigorously noted that this study can only demonstrate, to a certain extent, that students with relatively high anxiety early on generally exhibit a relatively weaker level of academic engagement in subsequent learning; it cannot prove that students' own academic engagement declines significantly over time. Furthermore, due to the limitations of a non-experimental longitudinal design, the combined effects of confounding variables—such as individual traits, classroom environment, and prior academic foundation—cannot be ruled out. Therefore, a stable temporal predictive relationship exists between the two variables.

This study further found that academic engagement cannot predict subsequent anxiety levels in the opposite direction. This differs from the conclusions of some cross-sectional studies, which suggest that increasing academic engagement can alleviate anxiety. The reason for this discrepancy is that cross-sectional studies cannot distinguish the temporal sequence of variables and tend to confuse the correlation between variables with a predictive relationship. In contrast, the longitudinal design of this study more objectively confirms that simply increasing academic engagement is unlikely to fundamentally improve adolescents' internalized anxiety. This finding also provides important guidance for school-based interventions. Academic development and psychological-emotional development are both independent of and closely interrelated. School education should not rely solely on academic tutoring to improve students' learning performance; instead, psychological and emotional counseling must be advanced in tandem with academic support, balancing the maintenance of students' mental health with the development of their academic abilities. This approach can mitigate the negative predictive impact of anxiety on adolescents' long-term academic adjustment.

### 4.3 Unidirectional positive prediction of psychological resilience on academic engagement

The longitudinal results of this study indicate that psychological resilience has a stable, unidirectional, and positive predictive effect on subsequent academic engagement, and that this positive association remains robust over time. This observed pattern is largely consistent with the core tenets of psychological capital theory and aligns with the mainstream conclusions of previous longitudinal studies on adolescents (Liang et al., 2026; Rich et al., 2023). As a crucial positive psychological resource, psychological resilience provides internal psychological support when students face academic pressure, test anxiety, and learning difficulties, helping them maintain relatively stable learning motivation and persistence; therefore, students who exhibit relatively high early psychological resilience tend to sustain positive learning attitudes and consistent task engagement in their long-term academic progression afterward. Compared to previous studies, which were predominantly cross-sectional or short-term two-wave follow-ups, this study further characterizes the long-term temporal relationship between the two variables using multi-wave longitudinal data, addressing the limitation of prior research in revealing long-term dynamic trends. It should be noted that this study only confirms the existence of a stable, positive temporal predictive relationship between the two variables; it does not imply that psychological resilience can actively catalyze or enhance academic engagement.

This study further found that prior academic engagement does not effectively predict subsequent psychological resilience. In terms of the longitudinal influence patterns of these variables, emotional traits and psychological protective resources exhibit strong stability over time and are not easily altered by short-term academic behaviors (Roberts and DelVecchio, 2000). Existing longitudinal studies generally confirm that, among adolescents, anxiety and psychological resilience primarily function as antecedent variables influencing academic engagement, rather than forming reverse predictive pathways (Marsh and Martin, 2011). For the junior high school students in this study, their levels of anxiety and psychological resilience are rooted in their upbringing and long-term psychological development; changes in academic engagement at a single stage are unlikely to produce lasting effects. This finding further clarifies the longitudinal boundaries of these three variables, demonstrates an asymmetric predictive relationship between them, and provides guidance for future intervention studies: simply increasing academic engagement cannot fundamentally improve adolescents' emotional states and psychological resilience.

At the same time, this study cannot completely rule out the combined influence of confounding variables—such as external support and a sense of academic achievement—on the relationship between the two variables. From a practical perspective, the temporal characteristics revealed in this study suggest that systematically cultivating students' psychological resilience resources can serve as an important reference for predicting and improving junior high school students' long-term academic engagement, providing continuous psychological support to help students cope with academic and psychological pressures throughout their junior high school years.

### 4.4 The longitudinal mediating role of psychological resilience

The temporal-separated mediation model in this study indicates that there is an inhibitory full mediation in the adaptation window from T1 to T2 for students in the first and second years of junior high school: anxiety at T1 reduces academic engagement only indirectly through psychological resilience at T2, and a weak positive direct path offsets the negative indirect effect; the positive and negative paths cancel each other out, ultimately resulting in inhibitory full mediation. This transmission pathway is consistent with short-term follow-up studies of new students and resource conservation theory (Mao et al., 2024); most previous studies reported mediation but failed to identify this offsetting effect, primarily due to the absence of a time-segmented longitudinal design. From T1 to T2, students had just transitioned to a new grade level and lacked sufficient reserves of psychological resilience; thus, anxiety could only affect learning by depleting psychological resources. The academic vigilance induced by short-term stress formed a small positive pathway, and the sequential measurement design also avoided confounding biases during this period.

However, during the T2 → T3 phase, the mediation chain breaks; anxiety at T2 no longer predicts resilience at T3, retaining only a direct negative effect on academic engagement. This change is consistent with long-term adolescent tracking studies and research on personality stability (Huttunen et al., 2024). Existing cross-sectional and two-wave studies generally assume that psychological resilience mediates the entire process; the discrepancy stems from the fact that most studies fail to distinguish between the adaptation and stabilization phases during grade transitions. After a period of adaptation, students' psychological resilience solidifies into a stable trait, and short-term anxiety struggles to alter its level; anxiety shifts from admission-related stress to long-term exam pressure, directly consuming cognitive resources and inducing academic avoidance; simultaneously, students develop fixed study habits, which, combined with adolescent emotional sensitivity, significantly amplify anxiety's direct inhibitory effect on learning.

In summary, the pathway through which anxiety influences academic engagement via psychological resilience is stage-specific. This finding partially addresses the static and one-sided limitations of cross-sectional studies and explains the inconsistencies in previous literature. However, this study can only demonstrate a temporal predictive relationship; it cannot rule out confounding variables such as family background, classroom atmosphere, and population differences, and thus cannot fully establish causality. Future research could employ intensive longitudinal tracking or intervention experiments to further validate the stage-specific mediating pattern.

### 4.5 Implications for educational practice

Cross-lagged correlation results show that anxiety is significantly negatively correlated with both psychological resilience and academic engagement, while psychological resilience positively predicts academic engagement. Based on these correlations, multi-tiered campus intervention measures can be implemented. Schools should conduct comprehensive anxiety screenings for all students each semester, offer regular group counseling sessions to alleviate academic anxiety caused by exams and interpersonal challenges, and incorporate mindfulness exercises and brief relaxation techniques into class meetings and lessons to promptly mitigate the negative impact of negative emotions on cognitive function and learning behavior. At the same time, a grade-level resilience development system should be established: in the first year of junior high, students should receive training in coping with setbacks as they adapt to the transition to secondary school; in the second year, guidance on positive attributions and a growth mindset should be provided in light of adolescent characteristics; and subject teachers should incorporate stress management education into daily contexts—such as homework feedback and test debriefings—to continuously strengthen students' protective psychological resources (Li et al., 2026). Furthermore, a home-school collaborative support mechanism has been established to regularly educate parents on appropriate stress-reduction methods, helping them avoid exacerbating their children's anxiety by placing excessive emphasis on academic performance. By comprehensively addressing both the management of negative emotions and the cultivation of positive psychological resources, a dual-pronged approach stabilizes junior high students' long-term academic engagement.

This study also found that the mediating pathways through which anxiety influences academic engagement exhibit stage-specific characteristics, enabling the implementation of tiered and differentiated campus mental health and academic support initiatives. During the school adjustment phase, students have limited psychological resilience reserves, and anxiety reduces academic engagement entirely by depleting these resources. Schools should therefore prioritize the cultivation of psychological resilience as a core intervention strategy by offering psychological adjustment courses for new students to teach emotion regulation and stress management skills, thereby promptly replenishing protective psychological resources and cutting off the indirect negative impact of anxiety on academic performance at its source; At the same time, this should be supplemented with counseling to address mild academic anxiety, thereby alleviating the stress caused by the transition to a new environment. As time passes and students gradually adapt to changes in grade level, psychological resilience no longer serves as a mediating factor. Routine academic anxiety can directly impair students' ability to concentrate on their studies, and cultivating psychological resilience alone cannot offset the negative effects of anxiety. At this stage, two types of interventions must be implemented simultaneously: on the one hand, routine academic anxiety counseling should be conducted, with group counseling sessions designed to alleviate pressure from exams and rankings, thereby reducing anxiety's encroachment on cognitive resources; on the other hand, students' psychological resilience should be continuously strengthened, relying on stable internal traits to buffer emotional fluctuations during adolescence, while incorporating guidance on study habits to reduce anxiety-induced behaviors such as procrastination and classroom avoidance (Zhu et al., 2026).

Overall, mental health education in elementary and secondary schools should not rely on a one-size-fits-all curriculum. Instead, intervention plans should be designed in a tiered manner based on the psychological mechanisms at play during the school adjustment period and the campus stabilization period, balancing emotional support with the cultivation of psychological resources to simultaneously safeguard students' mental health and ensure their long-term academic engagement.

### 4.6 Limitations and future directions

There are also some limitations in this research, which need to be addressed in the future. First, this study used self-report questionnaires to collect data. Response bias cannot be completely excluded. Future research can be combined with teacher evaluations, objective academic data, and other multi-source data, or experimental designs can be used to further verify the causal relationships. Second, the sample of this study came from only one middle school in eastern China. The sample focuses on the critical developmental period of the first and second years of junior high school, but its representativeness is limited. In the future, the sample range can be expanded to include different regions and different types of schools to improve the generalizability of the conclusions. Third, our conventional CLPM only captures between-group differences in variable levels. It lacks a latent variable structure to correct measurement error and cannot track individual fluctuations in anxiety and academic engagement over time, so it fails to verify whether one variable drives sustained shifts in the other. Future work may adopt LCLPM and latent change score models to address these two analytical limitations simultaneously. Fourth, the follow-up period in this study was only 6 months; future research could extend the follow-up period to cover the entire junior high school stage to explore the long-term dynamic trends of the variables. Fifth, this study used the total score of the full SCARED scale to represent students' overall anxiety; the scale includes anxiety dimensions unrelated to the school setting, such as separation anxiety and social phobia. The research results reflect the overall chain reaction of general anxiety on psychological resilience and academic engagement; they cannot distinguish the independent effects of school-related anxiety, separation anxiety, and social anxiety. Future studies focusing on specific dimensions could further break down different types of anxiety to clarify the differentiated impacts of anxiety inside and outside the school setting on adolescents' academic development, thereby addressing the analytical limitations arising from this study's focus on the overall construct.

## 5 Conclusions

With a three-wave longitudinal study design as the foundation, the current paper analyzed the dynamic temporal connections in a systematic manner and mediating effects among anxiety, psychological resilience, and academic engagement among junior secondary school students, yielding the following key conclusions. First, anxiety and psychological resilience exhibited bidirectional negative predictive effects during the early adaptation phase. In the later phase, only the protective effect of psychological resilience on anxiety persisted, with psychological resilience gradually developing into a stable protective resource. Second, anxiety exerted a stable, unidirectional negative predictive effect on academic engagement, acting as a precursor risk factor influencing academic engagement. Third, psychological resilience exerted a stable, unidirectional positive predictive effect on academic engagement, acting as an essential protective resource fostering academic engagement. Fourth, the longitudinal mediating effect of psychological resilience on the relationship between anxiety and academic engagement exhibits stage-specific differentiation: in the early stages, there is a fully mediating inhibitory effect; in the later stages of grade-level adaptation, the mediating chain disappears, leaving only the direct negative prediction of anxiety on academic engagement, with no longer any indirect effect mediated by psychological resilience.

## Data Availability

The original contributions presented in the study are included in the article/supplementary material, further inquiries can be directed to the corresponding author.
